# Transfusion: -80°C Frozen Blood Products Are Safe and Effective in Military Casualty Care

**DOI:** 10.1371/journal.pone.0168401

**Published:** 2016-12-13

**Authors:** Femke Noorman, Thijs T. C. F. van Dongen, Marie-Christine J. Plat, John F. Badloe, John R. Hess, Rigo Hoencamp

**Affiliations:** 1 Military Blood Bank, Ministry of Defense, Leiden, The Netherlands; 2 Ministry of Defense and Department of Trauma, Division of Surgery, University Medical Centre Utrecht, Utrecht, The Netherlands; 3 Expert Centre Force Health Protection, Ministry of Defense, Doorn, The Netherlands; 4 Transfusion Service, Harborview Medical Centre, Seattle, United States of America; 5 Ministry of Defense and Department of Surgery, Alrijne Medical Centre Leiderdorp, Leiden University Medical Centre, Leiden, the Netherlands; Massachusetts Institute of Technology, UNITED STATES

## Abstract

**Introduction:**

The Netherlands Armed Forces use -80°C frozen red blood cells (RBCs), plasma and platelets combined with regular liquid stored RBCs, for the treatment of (military) casualties in Medical Treatment Facilities abroad. Our objective was to assess and compare the use of -80°C frozen blood products in combination with the different transfusion protocols and their effect on the outcome of trauma casualties.

**Materials and Methods:**

Hemovigilance and combat casualties data from Afghanistan 2006–2010 for 272 (military) trauma casualties with or without massive transfusions (MT: ≥6 RBC/24hr, N = 82 and non-MT: 1–5 RBC/24hr, N = 190) were analyzed retrospectively. In November 2007, a massive transfusion protocol (MTP; 4:3:1 RBC:Plasma:Platelets) for ATLS^®^ class III/IV hemorrhage was introduced in military theatre. Blood product use, injury severity and mortality were assessed pre- and post-introduction of the MTP. Data were compared to civilian and military trauma studies to assess effectiveness of the frozen blood products and MTP.

**Results:**

No ABO incompatible blood products were transfused and only 1 mild transfusion reaction was observed with 3,060 transfused products. In hospital mortality decreased post-MTP for MT patients from 44% to 14% (P = 0.005) and for non-MT patients from 12.7% to 5.9% (P = 0.139). Average 24-hour RBC, plasma and platelet ratios were comparable and accompanying 24-hour mortality rates were low compared to studies that used similar numbers of liquid stored (and on site donated) blood products.

**Conclusion:**

This report describes for the first time that the combination of -80°C frozen platelets, plasma and red cells is safe and at least as effective as standard blood products in the treatment of (military) trauma casualties. Frozen blood can save the lives of casualties of armed conflict without the need for in-theatre blood collection. These results may also contribute to solutions for logistic problems in civilian blood supply in remote areas.

## Introduction

Deployed Medical Treatment Facilities (MTFs) need blood products for the treatment of casualties with severe blood loss. It is a challenge to provide sufficient blood products in an austere environment with unpredictable numbers and types of casualties, and long logistic lines. Besides regular transport of liquid stored erythrocyte concentrates (ECs) and -30°C Fresh Frozen Plasma (FFP), most countries use a walking blood bank for whole blood and platelet collections to provide for blood shortages and platelet needs. Although fresh whole blood is effective in the treatment of trauma casualties, it carries a risk of transmitting diseases or inducing graft versus host disease.[1–3] Furthermore availability of both products is dependent on the sufficient ABO blood type compatible pre-screened donors on site.[4, 5]

The Netherlands Armed Forces (NLAF) were the first to use mainly -80°C frozen blood products for the treatment of (military) casualties during deployments. In 2001 the Dutch Military Blood Bank (MBB) introduced -80°C Deep-frozen Thrombocyte Concentrate (DTC) and -80°C Deep-Frozen Plasma (DFP) in addition to red blood cells (RBCs) to replace whole blood in remote military theatres. In 2004 the NLAF MTFs (sea and land-based) became independent of regular ECs supply lines when the MBB implemented sterile processed -80°C Deep-frozen Erythrocyte Concentrates (DEC) that can be stored for 14 days after thaw and deglycerolization.[6–8]

During the armed conflict in Afghanistan between 2006–2016, the MBB provided 4 portable containerized Peripheral Blood Banks (PBBs) in MTFs with -80°C blood products, 3 PBBs served as a backup blood supply for hospitals operated by coalition forces (Canada/USA, United Kingdom and Germany). At the Multinational Base in Tarin Kowt (MBTK) a Role 2E (enhanced) MTF was operated by the NLAF from August 2006 to October 2010 and the total blood supply was provided by the MBB. The Dutch frozen blood supply system was also used by Australian and Singapore clinicians that were deployed in the Role 2E MTF at MBTK during that time.[9, 10]

Based on the observations that higher platelet and plasma use is associated with lower mortality rates in the military theatre,[11] clinical teams to be deployed were advised by the MBB to use the Dutch (frozen) blood products in a 4:3:1 RBC:DFP:DTC transfusion ratio when massive blood loss was anticipated (Massive Transfusion Protocol; MTP, implemented November 2007).

For this study the MBTK hemovigilance data were combined with retrospective patient data from the NLAF Battle Field Casualties database (BFCdb).[12–15]

Our first objective was to use these combined data to evaluate the use of -80°C blood products in the treatment of casualties. Our second objective was to assess the effect of the MTP with these products in the treatment of casualties.

## Methods

### Data collection

The Ministry of Defense (MOD) and the Institutional Review Board and Medical Ethics Committee of Leiden University, The Netherlands, approved this study.

To study the effect of frozen blood products and the 4:3:1 transfusion protocol on the treatment and outcome of trauma casualties, non-trauma casualties and trauma casualties that did not receive RBC during treatment were excluded from this analysis. Patient demographics, mechanism of injury, type of injury, injury severity score (ISS) and New Injury Severity Score (NISS), blood use, compatibility, transfusion reactions, length of stay (LOS) and 24-hour and in-hospital mortality was retrospectively collected from MBB hemovigilance data and the BFCdb, and combined for subsequent analysis.

### Blood products and transfusion policy

Blood products were produced by the MBB and thawed by the PBB according to international standards (Box 1).[6, 7] The frozen blood products have the advantage that they are fully tested, can be stored for a long time (DEC 10 years, DTC 4 years and DFP 7 years at least),[16–19] and thus allow for strategic stock management with ABO compatible blood products (O DEC, O DTC, AB DFP). Products are easily transported over long distances (dry-ice for 7 days, no electricity or re-icing required) and allow for flexible stock management in theatre to meet (sudden) demand and prevent spillage due to expiration. Limits are the time and consumables needed for pre-transfusion processing. To meet these limitations a pre-thawed liquid stock of RBCs and plasma is kept and next to the PBB, a separate air-conditioned container is used for consumables and backup frozen blood supply.

**Box 1: Detailed description of the frozen blood supply system Role 2E MTF at MBTK**

All blood products (whole blood, apheresis platelets, fresh frozen plasma (FFP) and liquid stored buffy coat depleted erythrocyte concentrates) were obtained from volunteer donors, fully tested and leukodepleted according to Council of Europe guidelines (Sanquin, The Netherlands). These ABO compatible products were purchased from Sanquin, processed and frozen to -80°C by the Military Blood Bank (MBB), and thawed and processed by the Peripheral Blood Bank (PBB, operated by MBB trained personnel as an extra task at the pharmacy or role 1 facility) according to international standards[6].

The PBB in Tarin Kowt was located in an air-conditioned mobile container with mechanical freezers (with CO2 back-up), 2–4 ACP^®^215s (automated cell processors to remove cryoprotectant glycerol from Deep-frozen Erythrocyte Concentrate [DEC],1 ACP215), 1–2 water baths, 1–2 sterile connection devices and sterile tube sealers. Another air conditioned mobile container was used for disposables and extra freezer(s).

Average PBB frozen stock levels were 60–140 DECs, 20–70 Deep-frozen Thrombocyte Concentrate (DTC) and 30–120 Deep-Frozen Plasma (DFPs) units. During these 4 years, on average every 2 weeks products were shipped from the MBB, and every 3 days DEC were washed (562/1583 days, max 5–6 units/ 8 hours/ ACP215 if on demand) at the PBB. RBC liquid/thawed stock level was thus maintained between 20–30 units. Generally ABO- compatible and Rhesus-D identical units red blood cells (RBCs) were issued based on cross matching or type and screen. In emergencies, RBC units issued were preferably O Rhesus-D negative (O-). However when O-stock was low, O+ RBC were preferably issued to male patients only. DFP was thawed on demand (35 minutes preparation time). Beginning of April 2009, the quality of 7 days 4°C stored thawed plasma (TDFP) was validated and the procedure was implemented by the MBB for Medical Treatment Facilities (MTFs) abroad. From that time 0–5 thawed DFP were kept at 4°C liquid stock. DTC were always prepared on demand (45 minutes preparation time).

Before the implementation of the Massive Transfusion Protocol on 1-11-2007 (Pre-MTP) blood products were ordered based on clinical experience and lab measurements: RBC if hemoglobin < 4/5 mmol/l, plasma transfusion if INR > 1.5 and platelet transfusion if platelet count < 100 10^9^/l in cases with Traumatic Brain Injury (TBI) or < 50 10^9^/l for non-TBI. After 1-11-2007 (post-MTP) the following MTP was advised: when ≥2-3L blood loss is suspected (ATLS^®^ class III-IV hemorrhage) start 4:3:1 RBC:Plasma:Platelets ratio until patient is hemodynamically stable, then, transfuse based on lab measurements.

This 4:3:1 ratio is based on the 1:1:1 RBC:Plasma:Platelet Rich Plasma (PRP) ratio which is equal to a 6:6:1 RBC:Plasma:Apheresis Platelets ratio. Because approximately only 70% of the frozen platelets is recovered after thaw and every thawed DTC is resuspended in one thawed DFP unit before use; this 6:6:1 ratio translates to the 4:3:1 ratio with DTC (±70%*6:6:1 = 4:4:1; corrected for plasma in DTC this becomes 4:3:1).

The MTP was a transfusion advice, not a package that was issued. To minimize impact of the first 1–2 RBC that may have been transfused before plasma and platelets were (ordered and) thawed, adherence to MTP protocol was defined as patients that were treated with more than 1:1.5 Plasma:RBC and/or 1:5 Platelets:RBC within 24 hours after arrival.

Before the MTP was implemented (pre-MTP), blood products were ordered based on clinical experience and lab measurements. After 1-11-2007 the MTP was implemented and a 4:3:1 ratio of RBC:Plasma:Platelet unit was advised for major blood loss. To minimize impact of the first 1–2 RBCs that may have been transfused before plasma and platelets were (ordered and) thawed, adherence to MTP protocol was defined as patients that were treated with more than 1:1.5 Plasma:RBC and/or 1:5 Platelets:RBC within 24 hours after arrival.

### Categories and statistics

Casualties were categorized for analysis based on RBC use during the first 24 hours after arrival in the Role 2E MTF at MBTK; massively transfused (MT) patients required transfusion of ≥ 6 RBC, and non-massively transfused (non-MT) patients required 1–5 units during the first 24 hours after arrival. For comparison with literature, casualties that received ≥ 10 RBC units/24hr were also analyzed as a separate group.

Data were analyzed using Microsoft Excel^®^ (Microsoft Corporation, 2014) and SPSS (Version 23, IBM Corporation, Armonk, New York). Chi-Square or Fisher's Exact Test were used to analyze qualitative categorical variables. Quantitative variables with normal distribution were analyzed using Students T-test. The Mann-Whitney U test was used for continuous data if distribution was not normal. Tests were two-sided for all cases, P < 0.05 was considered statistically significant.

## Results

### Patient demographics

Between August 2006 and October 2010 2,736 casualties were treated in the Role 2E MTF at MBTK. Of these casualties, 272 (9.9%) were in need of at least one RBC unit due to traumatic injuries. Most of the transfused trauma casualties were Afghan civilians and sustained penetrating and/or blast injuries from gunshots, mortars, improvised explosive devices (IED’s), rocket-propelled grenades or landmines (Table 1).

**Table 1:** General Patient demographics.

Category	Patients
*Subgroup*	*N = 272*
**Gender**	
*male*	91.2%
*female*	8.8%
**Age**	
*Adult (>16)*	73.9%
*child (<16)*	26.1%
**Nationality**	
*Afghanistan*	90.8%
*Netherlands*	6.3%
*Australia*	2.6%
*USA*	0.4%
**Force**	
*Local National*	66.2%
*Afghan National Security Forces*	22.1%
*Coalition Forces*	8.8%
*Other*	2.9%
**Mechanism of Injury**	
*Gunshot Wound*	41.2%
*Explosion*	43.4%
*Other* (*)	15.4%

(*) Other = Stabbing 3%, Accidents 11%, Burns 2%.

### Blood safety; compatibility and transfusion reactions

Out of 3,060 transfused blood products (2,400 frozen), one mild (urticarial) transfusion reaction was reported in the hemovigilance system, possibly due to platelet transfusion.

**Fig 1:**
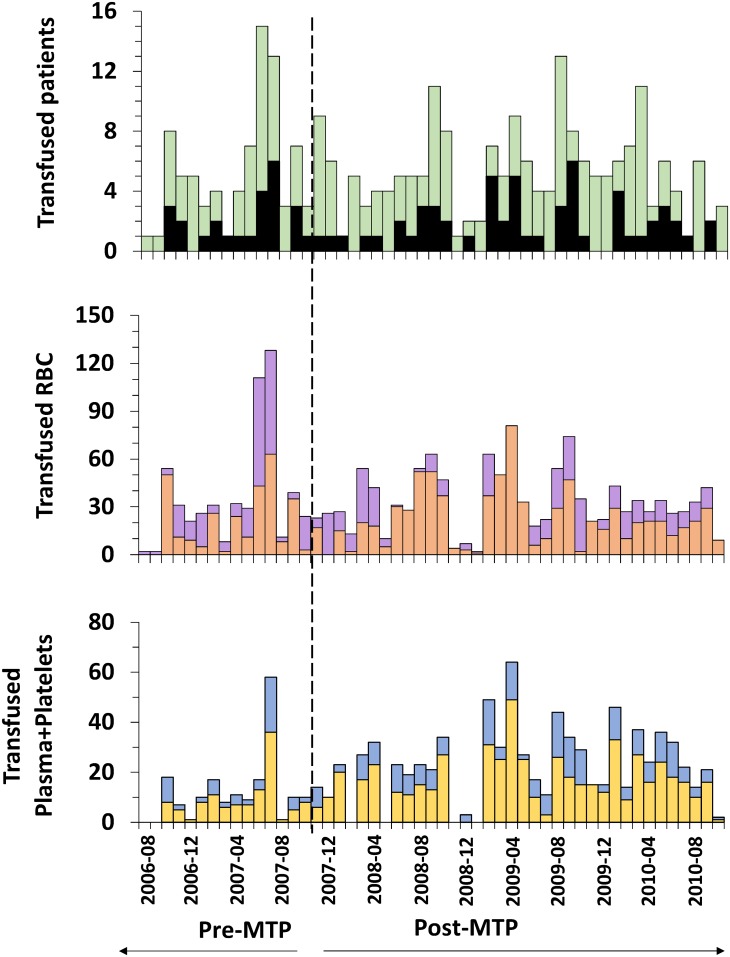
Transfused casualties and product use per month at MBTK. *MTBK* indicates Multinational Base Tarin Kowt, *RBC*; red blood cells, *MTP*: Massive Transfusion Protocol, *nr*: number, *MT*: massive transfusion, *EC*: Erythrocyte Concentrate. Upper panel: *black bars*: nr of MT patients, *green bars*: nr non-MT patients. Middle panel: *orange*: sum of thawed deglycerolized RBC units, *purple*: sum of EC units. Lower panel: *yellow*: sum of frozen plasma units, *blue*: sum of (plasma of) frozen platelets units.

Demand for blood products was unpredictable and directly related to the number of casualties (Fig 1). Nonetheless, all three product types were directly available for transfusion because of the frozen and pre-thawed (deglycerolized) liquid stock. The average age of the frozen products was approximately 1 year before thaw and transfusion (DEC 1.3±0.5 year; DFP 1.4±0.5 year and DTC 0.7±0.4 year). Only ABO compatible blood type units (O RBC, O Platelets and AB plasma) were shipped to Tarin Kowt, thus no ABO incompatible unit could be transfused. Few O+ RBC units were used for immediate transfusion to 6 Rhesus D- or unknown male casualties (S1 Table). Some O+ DTC in AB(+/-) plasma were transfused to 9 Rhesus D- or unknown male patients of which 5 MT patients that also received O+ RBC.

### Blood product use and patient outcome

Overall mortality was 12.5% (34/272). Although the type of injury was not different, the MT casualties were more severely wounded, had more abdominal and combined locations of injury and had a higher mortality rate (23.2%) compared to non-MT patients (7.9%) (S2 Table). Gender, Nationality and Force were not significantly different between the separate pre- and post-MTP, MT and non-MT comparison groups (data not shown). Injuries caused by explosions, number of wounds, relative percentage of poly-trauma, NISS and LOS increased while mortality decreased from 22.5% to 8.3% post-MTP (S3 Table).

**Fig 2:**
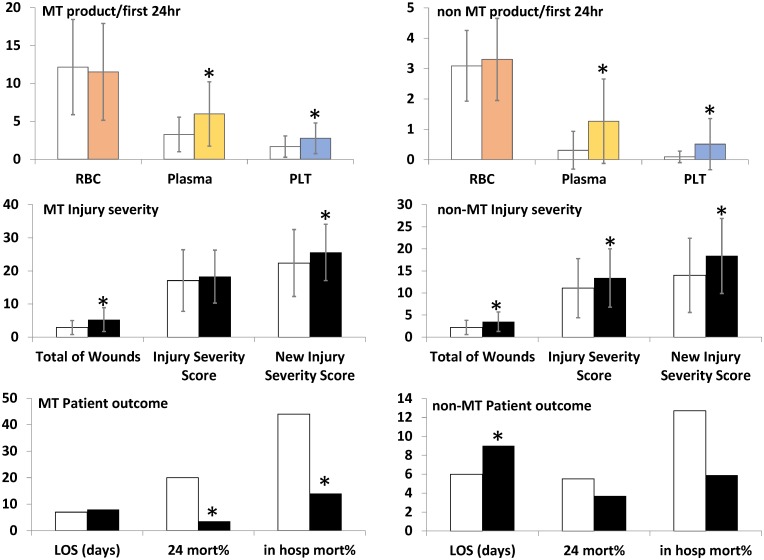
Transfusion, injury and outcome of MT and non-MT patients, pre- and post-MTP. *MT* indicates massive transfusion, *MTP*: Massive Transfusion Protocol, *RBC*: red blood cells; *Plasma*: frozen plasma; *PLT*: frozen platelets. *clear bars*: pre-MTP, *padded bars*: post-MTP, *: significantly different compared to pre-MTP.

After introduction of the MTP, average plasma and platelet use in both MT and non-MT patients increased significantly. Median blood use of MT patients was 10:3:2 RBC:Plasma:Platelets pre-MTP to 10:5:2 post-MTP, the latter approaching the 4:3:1 ratio. Median blood use of non-MT patients was 2:0:0 pre-MTP as well as post-MTP. Injury and LOS increased while mortality decreased in both transfusion categories (post-MTP MT 14%; P = 0.005; non-MT 5.9%; P = 0.139) (Fig 2 and S4 Table). When additional selection criteria were imposed (Table 2), the survival benefit of increased plasma and platelet use was most apparent for the severely injured massive bleeding patients.

**Table 2:** Effect of additional selection criteria on mortality statistics.

**Age>15 year N = 201**	**Pre-MTP**	**Post-MTP**	**P value**
24hr mortality	11% (6/57)	2% (3/144)	0.004 (†)
In hosp mortality	23% (13/57)	7% (10/144)	0.002 (‡)
**ISS>15 N = 111**	**Pre-MTP**	**Post-MTP**	**P value**
24hr mortality	23% (6/26)	6% (5/85)	<0.001 (†)
In-hosp mortality	50% (13/26)	15% (13/85)	0.001 (‡)
**ISS>15 N = 111**	**MT**	**Non-MT**	**P value**
24hr mortality	11% (6/53)	9% (5/58)	0.147 (†)
In-hosp mortality	30% (16/53)	17% (10/58)	0.083 (‡)
**ISS>15 N = 111**	**Pre-MTP**	**Post-MTP**	**P value**
MT 24hr mort.	29% (4/14)	5% (2/39)	0.004 (†)
MT in-hosp. mort.	64% (9/14)	18% (7/39)	0.002 (‡)
Non-MT 24hr mort.	17% (2/12)	7% (3/46)	0.253 (†)
Non-MT in-hosp. mort.	33% (4/12)	13% (6/46)	0.113 (‡)

Statistics: P values:

^†^ = Chi-Square test;

^‡^ = Fisher's Exact Test; <0.05 is considered significant.

*N* indicates number, *MTP*: Massive Transfusion Protocol, *hosp*: hospital, *ISS*: Injury Severity Score, *MT*: massive transfusion.

**Fig 3:**
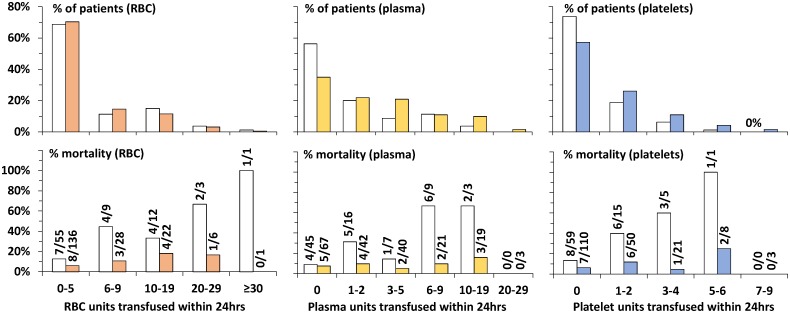
Transfusion and in-hospital survival per category of 24hr transfused blood products. *MT* indicates massive transfusion, *RBC*: red blood cells; *plasma*: frozen plasma; *platelets*: frozen platelets, *LOS*: length of stay. *clear bars*: pre-MTP, *padded bars*: post-MTP, *x/xx*: deceased/total number of patients.

The relation of increased mortality with increasing number of RBC, plasma or platelet units is more evident pre-MTP than post-MTP. Post-MTP RBC use was, not significantly, decreased (MT) and increased (not-MT), whereas platelet and plasma use significantly increased in both transfusion categories. As shown in Fig 3, mortality decreased post-MTP no matter which transfusion category, cut-off value or type of blood product.

Liquid stock of RBC consisting of deglycerolized thawed DEC and/or EC was maintained between 20–30 units. Average age of the RBC units was 65%-50% of their respective 4°C storage times (23±8 days EC; 7±4 days DEC). Significantly more casualties were transfused with DEC post-MTP (Fig 1 and S3 Table) because around that time the MBTK PBB’s capability to store and process DEC was doubled. In total 660 EC and 1,097 DEC were transfused. There were no differences in mortality between casualties that received 100% DEC (8/35 [23%] MT; 7/93 [7.5%] non-MT) or 100% EC (2/8 [25%] MT 4/53 [7.5%] non-MT).

**Fig 4:**
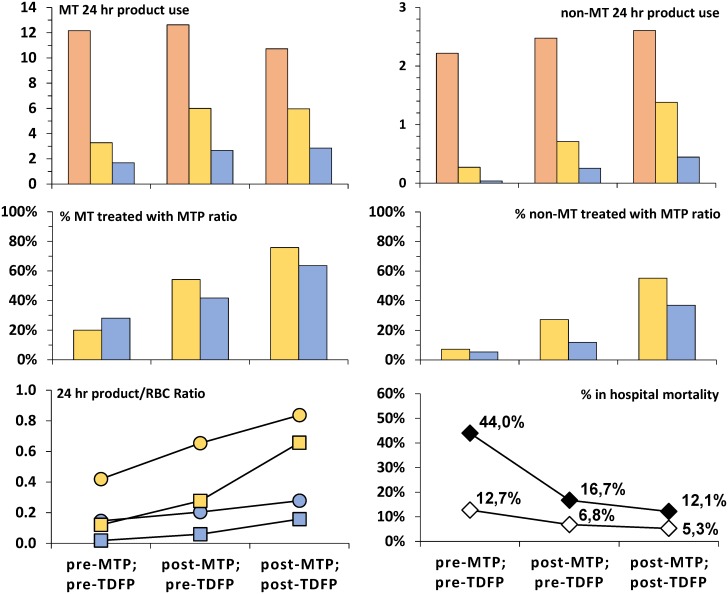
Introduction of TDFP, effect on blood product use and accompanying survival. *TDFP* indicates thawed deep frozen plasma, *MT*: massive transfusion, *MTP*: Massive Transfusion Protocol, *RBC*: red blood cell, *DTC*: deep frozen thrombocyte concentrate, *pts*: patients. Upper panel: *red* = RBC; *yellow* = frozen plasma; *blue* = frozen platelets. Middle panel: *yellow*: (plasma + plasma of DTC):RBC ≥1:1.5, *blue*: DTC:RBC ≥1:5. Lower panel left: *yellow*: (plasma + plasma of DTC)/RBC, *blue*: DTC/RBC, *circles*: MT pts, *squares*: non-MT pts. Lower panel right: *black diamonds*: MT pts, *white diamonds*: non-MT pts. Time frames during the period studied: pre-MTP; pre-TDFP: 08/2006–10/2007, N = 25 MT / 55 non-MT post-MTP; pre-TDFP: 11/2007–03/2009, N = 24 MT / 59 non-MT post-MTP; post-TDFP: 04/2009–10/2010, N = 33 MT / 76 non-MT.

After the introduction of 4°C stored thawed deep frozen plasma (TDFP) in April 2009, 498 TDFPs (1–5 units per patient) were transfused to 73% (24/33) of MT patients and 37% (28/76) of non-MT patients. Average age of TDFP was 4 days when transfused. Over time, median time between patient arrival and first DTC prepared on demand and issued, became shorter (for MT patients 4hr pre-MTP; 2hr post-MTP-pre-TDFP; 1hr post-MTP-post-TDFP and 14 to 5 to 2hr for non-MT patients). With the introduction of TDFP more casualties were treated with plasma and platelets and in accordance with the MTP protocol, while mortality rates declined (Fig 4).

Post-MTP more casualties got discharged either home or to a higher echelon of care (e.g. Role 3 MTF or out of theatre). LOS was related to the destination of casualties and shortest for casualties that were evacuated to a Role 3 MTF (S5 Table).

## Discussion

Between August 2006 and October 2010, 272 trauma casualties were transfused with predominantly frozen blood products. We showed for the first time that -80° frozen platelets and plasma in combination with (frozen) red blood cells can support an MTF in an austere environment with sufficient numbers of safe blood products for the treatment of unpredictable numbers of casualties without needing a walking blood bank capability.

### Principal findings

The frozen blood supply system and MTP has proven to be safe and effective during the Dutch deployment in Uruzgan, Afghanistan as shown by a low incidence of transfusion reactions, comparable blood use and post-MTP low mortality rates. Frozen red cells are now generally accepted,[8, 20] and besides The Netherlands, also used by the US and Czech Republic military.[21, 22]

In time, more but not all MT casualties were treated according to the MTP. This shows that the clinicians, instead of blindly implementing the 4:3:1 transfusion protocol, used this protocol together with their clinical judgment before ordering and/or transfusing blood products. With the introduction of the MTP more plasma and platelets were used and overall in-hospital mortality was significantly reduced from 22.5% pre-MTP to 8.3% post-MTP.

Frozen blood product use and product ratios were comparable, and mortality was low compared to that of trauma patients treated with similar numbers of standard blood products.

### Comparison with other studies

Data were compared to literature for (military) trauma patients and/or patients with massive blood loss between 2003–2015 (Table 3) and are discussed below.

**Table 3:** Comparison between current study and published data.

Study period	Type of hospital [Ref]	≥10rbc/24hr	Pat	Patient selection	Type of injury	ISS	24h Mort	Mort.	24hr RBC	Plasma /RBC	Plt/RBC	24hr Plt
		%	Nr			mean	%	%	mean	24hr mean	24hr mean	mean
2006–2007	trauma civilian[23]	100%	94	Pre-MTP 10:4:2 prp	30% pen., 70% blunt	30	-	66	20	0.6*	0.06*	1.1
2006–2007	trauma civilian[23]	100%	94	Post-MTP 10:4:2 prp	56% pen., 44% blunt	32	-	51	19	0.5*	0.03*	0.5
2007–2009	trauma civilian[24]	100%	132	Pre-MTP 12:12:1 aph plt	41% pen., 59% blunt	29	32	50	23	0.3	0.10	2.0
2007–2009	trauma civilian[24]	100%	132	Post-MTP 12:12:1 aph plt	49% pen., 51% blunt	27	29	48	24	0.5	0.10	2.0
**2006–2007**	**current study**	**100%**	**16**	**Pre-MTP 4:3:1 DTC**	**38% gsw, 50% expl**	**17**	**19**	**44** (φ)	**15**	**0.3 [0.4]**	**0.13 [0.09]**	**1.9**
2002–2003	civilian[25]	100%	390	Pre-MTP 5:5:2 bc plt	all departments	-	-	32	19	0.6*	0.06*	1.1
2005–2006	civilian[25]	100%	442	Post-MTP 5:5:2 bc plt	all departments	-	-	20	18	0.8*	0.19*	3.3
**2007–2010**	**current study**	**100%**	**29**	**Post-MPT 4:3:1 DTC**	**45% gsw, 45% expl**	**22**	**10**	**17** (φ)	**16**	**0.5 [0.8]**	**0.26 [0.18]**	**3.9**
**2006–2007**	**current study**	**20%**	**80**	**Pre-MTP Afghanistan MBTK**	**51% gsw, 33% expl**	**13**	**10**	**23** (φ)	**5**	**0.2 [0.2]**	**0.06 [0.04]**	**0.6**
2004–2007	combat JTTR[26]	35–40%	2,430	≥18 yr. Iraq (82% 2004–2007)	23% gsw, 72% expl	19 (m)	-	18	10	0.6	0.04 **	-
2008–2011	combat JTTR[26]	40–50%	1,202	≥18 yr. Afghanistan (91% 2008–11)	23% gsw, 74% expl	22 (m)	-	12	13	0.8	0.10 **	-
**2007–2010**	**current study**	**15%**	**192**	**Post-MTP Afghanistan MBTK**	**37% gsw, 48% expl**	**15**	**4**	**8** (φ)	**5**	**0.2 [0.6]**	**0.15 [0.11]**	**1.1**
		%	Nr			median	%	%	median	median	median	median
2003–2005	combat JTTR[27]	100%	31	Low plasma $ $	94% pen., 6% blunt	18	-	65 (φ)	16	0.1	-	0 (0.1#)
2004–2006	combat JTTR[28]	100%	214	No platelets, no fresh whole blood	93% pen., 7% blunt	20	35.9	57	14	0.5	0	0
2004–2006	combat JTTR[28]	100%	154	Medium platelet 1:16–1:8 aph plt:rbc $	94% pen., 6% blunt	21	13.2	40	17	0.7	0.12*	2
2003–2005	combat JTTR[27]	100%	53	Medium plasma $ $	94% pen., 6% blunt	18	-	34 (φ)	16	0.4	-	0 (1.0#)
2004–2006	combat JTTR[29]	100%	85	≥1 fresh whole blood, no aph plt $ $	92% pen., 8% blunt	26	18.8	34 (φ)	19	0.6*	0.13(#)	- (2.5#)
2004–2006	combat JTTR[29]	100%	284	≥1 apheresis platelets, no fwb $	93% pen., 7% blunt	22	15.8	25 (φ)	19	0.6*	0.11*	2
2004–2006	combat JTTR[28]	100%	96	High platelet ≥1:8 aph plt:rbc $	92% pen., 8% blunt	21	5.2	25	20	0.7	0.15*	3
2003–2005	combat JTTR[27]	100%	162	High plasma $ $	95% pen., 5% blunt	18	-	19 (φ)	17	0.7	-	0 (2.3#)
**2006–2010**	**current study**	**100%**	**21**	**≥1:5 DTC:rbc**	**52% gsw, 43% expl**	**17**	**19.0**	**19** (φ)	**12**	**0.5 [0.9]**	**0.31 [0.22]**	**4**
**2006–2010**	**current study**	**100%**	**23**	**≥1:1.5 (plasma + plasma DTC):rbc**	**52% gsw, 44% expl**	**17**	**17.0**	**17** (φ)	**12**	**0.6 [0.9]**	**0.30 [0.21]**	**4**
**2006–2007**	**current study**	**64%**	**25**	**Pre-MTP 4:3:1 DTC; ≥6 rbc/24hr**	**52% gsw, 40% expl**	**16**	**20.0**	**44** (φ)	**10**	**0.3 [0.4]**	**0.13 [0.09]**	**2**
2012–2013	trauma civilian[30]	47%	342	RCT MTP 12:12:1 (≥6 rbc/24hr)	51% pen., 49% blunt	26	17.0	26	9	0.6*	0.11*	1
2012–2013	trauma civilian[30]	45%	338	RCT MTP 6:6:1 (≥6 rbc/24hr)	46% pen., 54% blunt	27	12.7	22	9	0.8*	0.22*	2
**2007–2010**	**current study**	**51%**	**57**	**Post-MTP 4:3:1 DTC; ≥6 rbc/24hr**	**42% gsw. 49% expl**	**17**	**3.5**	**14** (φ)	**10**	**0.5 [0.8]**	**0.23 [0.16]**	**2**

Table is grouped by available data (average or median) % of patients receiving ≥ 10 RBC/24hr and type of patient selection. Groups are sorted by mortality rate. **Calculations**: **Plasma** = FFP = DFP | [**plasma**] = (DFP + Plasma of DTC) ratio | **Plt** = 1 * aphPLT = 1.5 * bcPLT = 6 * PRP = 2 * FWB = 1 * DTC | [**Plt**] = 1.4*DTC if 70% recovery is taken into account || If treatment with components in combination with FWB: **RBC** = RBC+FWB | **PLT** = aphPLT+0.5*FWB | **Plasma** = FFP + FWB.

MT indicates massive transfusion, MTP: Massive Transfusion Protocol, N: number, ISS: Injury Severity Score, JTTR: Joint Theatre Trauma Registry, RCT: Randomized Clinical Trial (age>15yr), pen: penetrating, gsw: gunshot wound, expl: explosions, 24hr Mort: 24-hour mortality, Mort: 28–30 days mortality, FWB: Fresh Whole Blood, RBC: Red Blood Cell units, PLT: platelets, aphPLT, Apheresis, liquid stored PLT in plasma, DTC: apheresis frozen PLT in plasma, bcPLT: PLT prepared from 4 buffy coats in Tsol^®^ storage solution, PRP: platelet rich plasma prepared from 1 FWB, FFP: Fresh Frozen Plasma (-30°C), DFP: Deep Frozen Plasma,

^(φ)^ = in-hospital mortality;

^(m)^ = median;

^(#)^ = mean;

^$^ = Role 3 MTF;

^$ $^ Role 3 MTF with nr of fwb and aph plts reported separately,[29] transformed to PLT according to above mentioned calculations.

* = if not reported, we calculated the ratio from average or median plasma or platelet transfusions/24rs divided by average or median RBC transfusions/24hr.

** = Platelet ratio is underestimated; fwb and aph plt were used, fwb were reported by authors as 0.116 aph plt transfusion instead of 0.5 aph plt transfusions.[26]

Although transfusion reactions may have been overlooked in trauma, it is unlikely that, if noted, those were not reported. Every product transfused was accompanied with a hemovigilance surveillance form, and communication between PBB and surgical team was not hampered by distance or unavailability of personnel. The incidence of a single transfusion reaction, 1 in 3,060 products, is low. This is probably related to the fact that with the frozen blood supply system, all products are leukodepleted and fully tested for pathogens prior to cryopreservation. Furthermore, no errors with blood type can be made (O+ and O- cellular products, AB(+/-) plasma), platelets are not stored after thaw for more than 6 hours (which reduces the chance of bacterial contamination),[31] few old liquid stored RBC were transfused and the plasma was from AB male donors only, all of which reduce the risk of transfusion reactions.[32, 33] No side effects other than normal reactions to fresh platelets have been reported since the introduction of frozen platelets for thrombocytopenic civilian patients in 1972.[34, 35] The average age of the RBC was below the 30–42 day threshold level of strong RBC storage lesion effects,[36, 37] which is one of the advantages of the frozen blood supply system. Both liquid stored and thawed RBC products were transfused at approximately half of the maximum storage time and RBC storage lesions are not likely to have affected patient outcome in the current study.

Generally, not surprisingly, casualties that received more RBC are more severely injured and thus have a lower chance of survival.[38–40] Although RBC use increases with injury severity and mortality, there is no defined RBC threshold that can be associated with a sudden increase in mortality.[38] Accordingly, no threshold could be detected in our study. It has been suggested that early transfusion with high transfusion rates reduces requirement for MT,[41] which may be the case in our study. To prevent bias, we chose the ≥6 RBC/24hr ratio in our assessment, as these categories represented ±30% of casualties pre- and post-MTP, whereas fewer post-MTP casualties were treated with ≥10 RBC/24hr.

Compared to civilian studies, most military studies (including current study) report similar 24-hour RBC use for treatment of casualties with lower injury severity scores. Accordingly, compared to the civilian randomized clinical trial with 6:6:1 RBC:Plasma:Platelets (issued in packages in early treatment),[30] MT patients in the current study had a lower ISS and similar 24-hour RBC, plasma and platelet use, while (24hr) mortality was low. This is probably related to the different cause and type of injuries (50–70% blunt versus >84% penetrating and explosions).

The patients selected for the randomized clinical trial were those with severe bleeding, age ≥15 and requiring the highest level of trauma care.[30] When only casualties with ISS >15 instead of MT-patients were compared to these data, post-MTP (24hr) median ISS was the same and (24hr) mortality was still 7% lower. This is probably related to the different patient demographics and combat protection and care strategies.[42]

In the Joint Theatre Trauma Registry (JTTR) 2003–2012 analysis,[26] only US military casualties with an age ≥18 were included while many children (26% age<16) were included in the current study. The exclusion of children from analysis did not result in higher mortality rates. The (surgical) treatment of children and casualties with major hemorrhage, admitted to the Role 2E MTF at MBTK, is described in more detail elsewhere.[12, 13]

IED exposure and type of injury of casualties presented at MBTK was similar to that of coalition forces operating during the same period.[15, 43] ISS, % of explosions and blood product use was lower and mortality was 4% lower compared to JTTR data of Afghanistan.[26] When only MBTK patients with ISS>15 were compared, median ISS was similar and post-MTP mortality was 5% higher. This may be related to the relative high percentage of civilian casualties (66% overall) transfused at MBTK, who did not have the same access to protective measures and immediate combat care.

Similar to our study, the JTTR data show that over the years, ISS increased while mortality rates decreased, which was accompanied with a higher plasma and platelet use.[26, 44] The decreased mortality was mainly attributed to the increased platelet ratios that was highly associated with reduced mortality rates, while high plasma ratios had an additive effect.[26] Compared to other JTTR studies that retrospectively studied blood product ratios, (24hr) mortality of patients transfused with ≥10 RBC/24hr at MBTK was lower with higher frozen platelet and plasma/24hr RBC ratios and lower numbers of RBC transfused/24hr.

### Possible explanations

Since in the Role 2E MTF at MBTK each unit of frozen platelets was resuspended after thaw in fresh thawed plasma, high platelet ratios were always accompanied with high plasma ratios. In our study and other military and civilian studies, mortality of MT patients was lowest with a plasma ratio ≥0.7 in combination with a platelet ratio ≥0.15. These cut off values are similar to the in 2003 calculated predicted optimal transfusion ratio to prevent dilutional coagulopathy (±2 plasma/3 RBC and ±1.3 apheresis platelet/10 RBC).[45] To correct dilutional and/or consumptive coagulopathy, more plasma and platelets may be required.

DTCs contain a higher percentage of activated platelets and microparticles, and appear to have a higher (in vitro) hemostatic capacity compared to liquid stored platelets.[19, 34, 46–50] Coagulopathic trauma patients who have reduced hemostatic capacity need more RBC transfusions and have reduced concentrations of platelet derived microparticles.[51, 52] Frozen platelets may be extra effective in bleeding patients because the product contains activated platelets and platelet derived microparticles that can restore the above mentioned reduced hemostatic capacity.[46]

The in vivo clearance of activated previously frozen platelets is more rapid,[53, 54] which could be a disadvantage when platelets are transfused for prophylactic purposes, but may be an advantage in the acute treatment of bleeding trauma patients. Frozen platelets are given to trauma patients during the early coagulopathic bleeding phase. The activated platelets and microparticles have probably disappeared from the circulation before the onset of the pro-thrombotic phase which can be an advantage because higher concentrations of platelets are associated with thromboembolic events.[55–57]

### Limitations of the study

Limitations of this study are inherent to its retrospective nature and available data. Several unknown factors such medical evacuation times from injury to MBTK, might have changed over time and possibly influenced the pre- and post-MTP mortality rates. Also cause of death, pre-hospital treatment and post-hospital outcome are unknown factors in this study. Most likely, because of the nature, time-frame and duration of the conflict, the experience of the deployed clinicians with damage control surgery and resuscitation increased during 2006–2010. Therefore, future research in which these possible confounding factors and long-term outcomes can be taken into account, should be undertaken to confirm our results.

### Future studies

The push for introduction of drones for transport of blood indicates a demand for a readily available blood supply system in civilian settings also.[58] The US military practice is to resupply 2 times a week.[21] This system requires additional walking blood banks to provide for platelets and red cell shortages with whole blood,[21, 59] which is not easily introduced in civilian settings.[60] Frozen blood products are more expensive to produce but may be cheaper to use when the reduced outdating, reduced logistics, and no longer required (safety) costs of a walking blood supply system are taken into account. However, this remains to be demonstrated in future cost analysis and blood management studies.

Our results and shared experience at MBTK, led the Australian Defense Force to start implementing a frozen blood supply system in collaboration with the Australian Red Cross.[7, 48, 61] Current research (sponsored by the US and Australian military) focuses on in vivo recovery, effectivity and safety of frozen platelets in thrombocytopenic patients and elective surgery,[54, 61–63] which may result in a more general acceptation of -80°C frozen platelets for the benefit of military and civilian (trauma) casualties worldwide.

## Conclusions

Fresh frozen products are at least equally effective and safer to use compared to blood products used by standard military blood banks that are more dependent on logistics and require untested/not fully tested ABO compatible whole blood or apheresis donations from their deployed military personnel.

A frozen blood supply is less dependent on regular long distance product logistics and facilitates both strategic and on-site stock management. The frozen blood products are ABO compatible, leukodepleted and fully tested for transmittable diseases. Compared to civilian and military treatment facilities that implemented MTP, blood use of massively transfused trauma casualties was similar and mortality was low.

The frozen blood supply system combined with the 4:3:1 massive transfusion protocol has contributed to the survival of combat casualties and may also be a safe and effective provision for civilian trauma centers and blood banks during (un)expected blood shortages. Future clinical research should be undertaken to confirm our results in a civilian trauma setting.

## Supporting Information

S1 Table Patient RhD and ABO RhD blood type of transfused blood products. *RhD* indicates Rhesus D, *pat*: patients, *n*: number, *prod*: products, *RBC*: Red Blood Cell units, *DTC*: Deep-frozen Thrombocyte Concentrate units, *DFP*: Deep-frozen Plasma units. (*) transfused in 2 MT RhD negative male patients, (**) transfused in 3 MT and 1 non-MT male patient, ABO-RhD unknown to PBB. Note: Patient ABO type was 11% AB, 26% B, 25% A, 32% O, 7% unknown.
(DOCX)

S2 Table Patient demographics MT and non-MT patients. *MT* indicates massive transfusion, *Sign*: significance, *N*: number, *NS*: not significant, *ISS*: Injury Severity Score, *NISS*: New Injury Severity Score, *LOS*: Length of Stay. ± standard deviation (median); P values: † = Chi-Square test; ‡ = Fisher's Exact Test; # = T-Test; * = Mann Whitney U Test.
(DOCX)

S3 Table Patient demographics pre- and post-MTP. *MTP* indicates Massive Transfusion Protocol, *N*: number, *NS*: not significant, *Sign*: significance, *ISS*: Injury Severity Score, *NISS*: New Injury Severity Score, *MT*: massive transfusion, *RBC*: Red Blood Cell units, *EC*: Erythrocyte Concentrate units, *DEC*: Deep-frozen Erythrocyte units, *LOS*: Length of Stay. Plasma(+) = plasma units + plasma present in DTC (1 unit plasma/DTC). Average ± standard deviation (median); P values: * = Mann Whitney U Test; † = Chi-Square test; ‡ = Fisher's Exact Test; # = T-Test.
(DOCX)

S4 Table Patient demographics, blood use MT and non-MT pre- and post-MTP. *MT* indicates massive transfusion, *MTP*: Massive Transfusion Protocol, *N*: number, *NS*: not significant, *N/A*: not applicable, *ISS*: Injury Severity Score, *NISS*: New Injury Severity Score, *RBC*: Red Blood Cell units, *EC*: Erythrocyte Concentrate units, *DEC*: Deep-frozen Erythrocyte units, *LOS*: Length of Stay. Plasma(+) = plasma units + plasma present in DTC (1 unit plasma/DTC). Average ± standard deviation (median); P values: † = Chi-Square test; ‡ = Fisher's Exact Test; # = T-Test; * = Mann Whitney U Test.
(DOCX)

S5 Table Survivors; length of stay at MBTK and destination after treatment. *MBTK* indicates Multinational Base Tarin Kowt, *MT*: massive transfusion, *Sign*: significance, *MTP*: Massive Transfusion Protocol, *LOS*: Length of Stay, *N*: number, *NS*: not significant, *N/A*: not applicable, *int*: international (out of theatre), *Hero*: camp Hero in Kandahar, *ANA*: Afghan National Army, *MTF*: Medical Treatment Facility, *hosp*: hospital, *TK*: Tarin Kowt, *KAF*: Kandahar Airfield. Total number of survivors = 238; subgroup n = 217: excl. n = 21 discharge destination or LOS not registered in BFCdb; subgroup n = 227: excl. n = 11 discharge destination not registered in BFCdb. Average ± standard deviation (median); p-values: † = Chi-Square Test; * = Mann Whitney U Test.
(DOCX)
