# Supplementary material for: Transfusion: -80°C Frozen Blood Products Are Safe and Effective in Military Casualty Care
Source: PLoS One. 2016 Dec 13;11(12):e0168401. doi: 10.1371/journal.pone.0168401 (PMC5154589; doi:10.1371/journal.pone.0168401)
Supplement: S1 Table — RhD indicates Rhesus D, pat: patients, n: number, prod: products, RBC: Red Blood Cell units, DTC: Deep-frozen Thrombocyte Concentrate units, DFP: Deep-frozen Plasma units. (*) transfused in 2 MT RhD negative male patients, (**) transfused in 3 MT and 1 non-MT male patient, ABO-RhD unknown to PBB. Note: Patient ABO type was 11% AB, 26% B, 25% A, 32% O, 7% unknown. (DOCX) [file pone.0168401.s001.docx]

| **S1 Table. Patient RhD and ABO RhD blood type of transfused blood products.** | | | | | | |
| --- | --- | --- | --- | --- | --- | --- |
| **Transfused Products (n)** | **Tot pat** N=272 (100%) | **D+ pat** N=233 (86%) | **D- pat**  N=21  (7.7%) | **D? pat** N=18 (6.6%) | **% prod.**  N=3060  (100%) |  |
| RBC O+ | 908 | 889 | 3^(^*^)^ | 16^(^**^)^ | 30% |  |
| DTC O+ | 175 | 151 | 12 | 12 | 6% |  |
| RBC O- | 843 | 679 | 105 | 59 | 28% |  |
| DTC O- | 145 | 135 | 7 | 3 | 5% |  |
| DFP AB+/- | 989 | 895 | 59 | 35 | 32% |  |
| **Sum** | 3060 | 2749 | 186 | 125 | 100% |  |
