# Supplementary material for: Transfusion: -80°C Frozen Blood Products Are Safe and Effective in Military Casualty Care
Source: PLoS One. 2016 Dec 13;11(12):e0168401. doi: 10.1371/journal.pone.0168401 (PMC5154589; doi:10.1371/journal.pone.0168401)
Supplement: S2 Table — MT indicates massive transfusion, Sign: significance, N: number, NS: not significant, ISS: Injury Severity Score, NISS: New Injury Severity Score, LOS: Length of Stay. ± standard deviation (median); P values: † = Chi-Square test; ‡ = Fisher's Exact Test; # = T-Test; * = Mann Whitney U Test. (DOCX) [file pone.0168401.s002.docx]

| **S2 Table. Patient demographics MT and non-MT patients.** | | | |
| --- | --- | --- | --- |
| **Category** | **MT** | **Non-MT** | **Sign.** |
| *Subgroup* | *N=82* | *N=190* | *P-value* |
| **Age** | 26 ± 12 | 22 ± 12 | <0.01 (#) |
| *child (<16)* | 15.9% | 30.5% | <0.05 (†) |
| **Mechanism of injury** |  |  | NS (†) |
| *Gunshot Wound* | 45.1% | 39.5% |  |
| *Explosion* | 46.3% | 42.1% |  |
| *other* | 8.5% | 18.4% |  |
| **Location of Injury** |  |  | <0.05 (†) |
| *‎Head/Neck* | 0.0% | 1.6% |  |
| *Thorax* | 3.7% | 5.3% |  |
| *Abdomen* | 14.6% | 9.0% |  |
| *Extremities* | 24.5% | 38.0% |  |
| *External* | 0.0% | 4.8% |  |
| *Combined* | 57.3% | 41.3% |  |
| **Injury severity** |  |  |  |
| *Total of Wounds* | 4.5 ± 3.4 (3) | 3.1 ± 2.2 (2) | <0.01 (*) |
| *ISS* | 17.9 ± 8.4 | 12.7 ± 6.7 | <0.01 (#) |
| *NISS* | 24.7 ± 9.1 | 17.1 ± 8.7 | <0.01 (#) |
| **LOS and Survival** |  |  |  |
| *LOS(days)* | 8±10 (4) | 8±11 (5) | NS (*) |
| *24hr Mortality* | 8.5% | 4.2% | <0.01 (†) |
| *In hospital Mortality* | 23.2% | 7.9% | <0.01 (‡) |
