# Supplementary material for: Transfusion: -80°C Frozen Blood Products Are Safe and Effective in Military Casualty Care
Source: PLoS One. 2016 Dec 13;11(12):e0168401. doi: 10.1371/journal.pone.0168401 (PMC5154589; doi:10.1371/journal.pone.0168401)
Supplement: S3 Table — MTP indicates Massive Transfusion Protocol, N: number, NS: not significant, Sign: significance, ISS: Injury Severity Score, NISS: New Injury Severity Score, MT: massive transfusion, RBC: Red Blood Cell units, EC: Erythrocyte Concentrate units, DEC: Deep-frozen Erythrocyte units, LOS: Length of Stay. Plasma(+) = plasma units + plasma present in DTC (1 unit plasma/DTC). Average ± standard deviation (median); P values: * = Mann Whitney U Test; † = Chi-Square test; ‡ = Fisher's Exact Test; # = T-Test. (DOCX) [file pone.0168401.s003.docx]

| **S3 Table. Patient demographics pre- and post-MTP.** | | | |
| --- | --- | --- | --- |
| **Category** | **pre-MTP** | **post-MTP** | **Sign.** |
| *Subgroup* | *N=80* | *N=192* | *P-value* |
| **Age** | 22 ± 13 | 23 ± 12 | NS (#) |
| *child (<16)* | 28.7% | 25.0% | NS (‡) |
| **Mechanism of Injury** |  |  | <0.05 (†) |
| *Gunshot Wound* | 51.2% | 37.0% |  |
| *Explosion* | 32.5% | 47.9% |  |
| *Other* | 16.3% | 15.2% |  |
| **Location of Injury** |  |  | <0.01 (†) |
| *Head‎/Neck* | 1.3% | 1.0% |  |
| *Thorax* | 2.5% | 5.8% |  |
| *Abdomen* | 20.0% | 6.8% |  |
| *Extremities* | 37.6% | 32.4% |  |
| *External* | 6.3% | 2.1% |  |
| *Combined* | 32.5% | 51.8% |  |
| **Injury Severity** |  |  |  |
| *Total of Wounds* | 2.4 ± 1.8 (2) | 4.0 ± 2.8 (3) | <0.01 (*) |
| *ISS* | 13 ± 8.1 | 14.8 ± 7.4 | NS (#) |
| *NISS* | 16.7 ± 9.8 | 20.5 ± 9.1 | <0.01 (#) |
| *%MT patients* | 31.3% | 29.7% | NS (‡) |
| **24hr Blood transfusion** |  |  |  |
| *RBC* | 5.3 ± 5.9 (2) | 5.2 ± 5.5 (4) | NS (*) |
| *Plasma* | 1.2 ± 2 (0) | 2.5 ± 3.4 (2) | <0.01 (*) |
| *Platelets* | 0.6 ± 1.1 (0) | 1.1 ± 1.7 (0) | <0.01 (*) |
| *Platelet /RBC ratio* | 0.06 ± 0.12 (0) | 0.15 ± 0.23 (0) | <0.01 (*) |
| *Plasma(+)* | 1.8 ± 2.9 (0) | 3.6 ± 4.8 (2) | <0.01 (*) |
| *Plasma(+)/RBC ratio* | 0.2 ± 0.3 (0) | 0.6 ± 0.5 (0.4) | <0.01 (*) |
| **Total Frozen or Liquid RBC** |  |  |  |
| *Liquid EC* | 3.2 ± 5 (1.5) | 2.1 ± 3.8 (0) | <0.05 (*) |
| *# Deep Frozen EC* | 3.7 ± 4.8 (2) | 4.2 ± 5.2 (2) | NS (*) |
| *Percentage DEC/RBC* | 52 ± 43 (50) | 66 ± 40 (100) | <0.05 (*) |
| **LOS and Survival** |  |  |  |
| *LOS (days)* | 6±8 (4) | 9±12 (5) | NS (*) |
| *24hr Mortality* | 10.0% | 3.6% | <0.01 (‡) |
| *In hospital Mortality* | 22.5% | 8.3% | <0.01 (‡) |
