# Supplementary material for: Transfusion: -80°C Frozen Blood Products Are Safe and Effective in Military Casualty Care
Source: PLoS One. 2016 Dec 13;11(12):e0168401. doi: 10.1371/journal.pone.0168401 (PMC5154589; doi:10.1371/journal.pone.0168401)
Supplement: S4 Table — MT indicates massive transfusion, MTP: Massive Transfusion Protocol, N: number, NS: not significant, N/A: not applicable, ISS: Injury Severity Score, NISS: New Injury Severity Score, RBC: Red Blood Cell units, EC: Erythrocyte Concentrate units, DEC: Deep-frozen Erythrocyte units, LOS: Length of Stay. Plasma(+) = plasma units + plasma present in DTC (1 unit plasma/DTC). Average ± standard deviation (median); P values: † = Chi-Square test; ‡ = Fisher's Exact Test; # = T-Test; * = Mann Whitney U Test. (DOCX) [file pone.0168401.s004.docx]

| **S4 Table. Patient demographics, blood use MT and non-MT pre-and post-MTP.** | | | | | | |
| --- | --- | --- | --- | --- | --- | --- |
| **Category** | **MT** | | | **non-MT** | | |
| *Subgroup* | ***pre-MTP***  *N=25* | ***post-MTP***  *N=57* | ***P value***  *MT* | ***pre-MTP*** *N=55* | ***post-MTP*** *N=135* | ***P value***  *Non-MT* |
| **Age** | 30 ± 13 | 24 ± 11 | NS (#) | 18.3 ± 11 | 23 ± 12 | <0.05 (#) |
| *child (<16)* | 4.0% | 21.1% | NS (‡) | 40.0% | 26.7% | NS (‡) |
| **Mechanism of Injury** |  |  | NS (†) |  |  | <0.05 (†) |
| *Gunshot Wound* | 52.0% | 42.1% |  | 50.9% | 34.8% |  |
| *Explosion* | 40.0% | 49.1% |  | 29.1% | 47.4% |  |
| *Other* | 8.0% | 8.8% |  | 20% | 17,8% |  |
| **Location of Injury** |  |  | NS (†) |  |  | <0.01 (†) |
| *Head‎/Neck* | 0.0% | 0.0% |  | 1.8% | 1.5% |  |
| *Thorax* | 4.0% | 3.5% |  | 1.8% | 6.7% |  |
| *Abdomen* | 24.0% | 10.5% |  | 18.2% | 5.2% |  |
| *Extremities* | 20.0% | 26.4% |  | 45.5% | 35.0% |  |
| *External* | 0.0% | 0.0% |  | 9.1% | 3.0% |  |
| *Combined* | 52.0% | 59.6% |  | 23.6% | 48.5% |  |
| **Injury Severity** |  |  |  |  |  |  |
| *Total of Wounds* | 2.9 ± 2.1 (3) | 5.3 ± 3.6 (4) | <0.01 (*) | 2.2 ± 1.6(2) | 3.5 ± 2.2 (3) | <0.01 (*) |
| *ISS* | 17.1 ± 9.3 | 18.3 ± 8.0 | NS (#) | 11.1 ± 6.7 | 13.4 ± 6.6 | <0.05 (#) |
| *NISS* | 22.4 ± 10.1 | 25.6 ± 8.5 | NS (#) | 14.0 ± 8.4 | 18.4 ± 8.5 | <0.01 (#) |
| **24hr Blood transfusion** |  |  |  |  |  |  |
| *RBC* | 12.2 ± 6.3 (10) | 11.5 ± 6.4 (10) | NS (*) | 2.2 ± 1.2 (2) | 2.6 ± 1.4 (2) | NS (*) |
| *Plasma* | 3.3 ± 2.3 (3) | 6.0 ± 4.2 (5) | <0.01 (*) | 0.3 ± 0.6 (0) | 1.1 ± 1.4 (0) | <0.01 (*) |
| *Platelets* | 1.7 ± 1.4 (2) | 2.8 ± 2.0 (2) | <0.05 (*) | 0.0 ± 0.2 (0) | 0.4 ± 0.8 (0) | <0.01 (*) |
| *Platelet /RBC ratio* | 0.15 ± 0.12(0.13) | 0.25 ± 0.16(0.23) | <0.01 (*) | 0.02 ± 0.11(0) | 0.10 ± 0.24(0) | <0.01 (*) |
| *Plasma(+)* | 5.0 ± 3.2 (5) | 8.8 ± 5.6 (8) | <0.01 (*) | 0.3 ± 0.6 (0) | 1.5 ± 1.8 (1) | <0.01 (*) |
| *Plasma(+)/RBC ratio* | 0.4 ± 0.3 (0.4) | 0.8 ± 0.3 (0.8) | <0.01 (*) | 0.1 ±0.3 (0) | 0.5 ± 0.5 (0.4) | <0.01 (*) |
| **In hospital transfusion** |  |  |  |  |  |  |
| *RBC* | 15.2 ± 7.2 (14) | 13.3 ± 8.0 (11) | NS (*) | 3.1 ± 2.4 (2) | 3.3 ± 2.2 (3) | NS (*) |
| *Plasma* | 4.0 ± 2.6 (4) | 6.7 ± 5.1 (6) | <0.05 (*) | 0.3 ± 0.7 (0) | 1.3 ± 1.6 (0) | <0.01 (*) |
| *Platelets* | 2.2 ± 2.1 (2) | 3.3 ± 2.4 (3) | <0.05 (*) | 0.1 ± 0.6 (0) | 0.5 ± 1.3 (0) | <0.01 (*) |
| *# Plasma(+)* | 6.2 ± 4.1 (6) | 10.0 ± 6.7 (9) | <0.05 (*) | 0.4 ± 0.8 (0) | 1.8 ± 2.3 (1) | <0.01 (*) |
| **MTP Plasma(+):RBC** |  |  |  |  |  |  |
| *<1:1.5Plasma(+):RBC 24hr* | 80.0% | 33.3% | <0.01 (†) | 92.7% | 57% | <0.01 (†) |
| *≥1:1.5Plasma(+):RBC 24hr* | 20.0% | 66.7% |  | 5.5% | 40% |  |
| *N/A: RBC24h = 0* | 0.0% | 0.0%) |  | 1.8% | 3% |  |
| **MTP platelet:RBC** |  |  |  |  |  |  |
| *<1:5 Platelet:RBC 24hr* | 72.0% | 45.6% | <0.05 (†) | 94.5% | 74.1% | <0.01 (†) |
| *≥1:5 Platelet:RBC 24hr* | 28.0% | 54.4% |  | 3.6% | 23.0% |  |
| *N/A: RBC24h = 0* | 0.0% | 0.0% |  | 1.8% | 3.0% |  |
| **Total Frozen / Liquid RBC** |  |  |  |  |  |  |
| *Liquid EC* | 7.2 ± 7.0 (6) | 4.2 ± 6.0 (2) | <0.05 (*) | 1.4 ± 2.1 (1) | 1.2 ± 1.8 (0) | NS (*) |
| *# Deep Frozen EC* | 8.0 ± 6.2 (8) | 9.1 ± 6.9 (8) | NS (*) | 1.7 ± 2.0 (2) | 2.1 ± 2.0 (2) | NS (*) |
| *Percentage DEC/RBC* | 55 ± 37 (45) | 69 ± 36 (83) | NS (*) | 50 ± 45 (50) | 65 ± 42 (100) | <0.05 (*) |
| **LOS and Survival** |  |  |  |  |  |  |
| *LOS (days)* | 7±7 (6) | 8±12 (3.5) | NS (*) | 6±9 (4) | 9±12 (6) | <0.05 (*) |
| *24hr Mortality* | 20.0% | 3.5% | <0.01 (†) | 5.5% | 3.7% | NS (†) |
| *In hospital Mortality* | 44.0% | 14.0% | <0.01 (‡) | 12.7% | 5.9% | NS (‡) |
