# Supplementary material for: Transfusion: -80°C Frozen Blood Products Are Safe and Effective in Military Casualty Care
Source: PLoS One. 2016 Dec 13;11(12):e0168401. doi: 10.1371/journal.pone.0168401 (PMC5154589; doi:10.1371/journal.pone.0168401)
Supplement: S5 Table — MBTK indicates Multinational Base Tarin Kowt, MT: massive transfusion, Sign: significance, MTP: Massive Transfusion Protocol, LOS: Length of Stay, N: number, NS: not significant, N/A: not applicable, int: international (out of theatre), Hero: camp Hero in Kandahar, ANA: Afghan National Army, MTF: Medical Treatment Facility, hosp: hospital, TK: Tarin Kowt, KAF: Kandahar Airfield. Total number of survivors = 238; subgroup n = 217: excl. n = 21 discharge destination or LOS not registered in BFCdb; subgroup n = 227: excl. n = 11 discharge destination not registered in BFCdb. Average ± standard deviation (median); p-values: † = Chi-Square Test; * = Mann Whitney U Test. (DOCX) [file pone.0168401.s005.docx]

| **S5 Table. Survivors; length of stay at MBTK and destination after treatment.** | | | | | | |
| --- | --- | --- | --- | --- | --- | --- |
| **Category** | **MT** | **non-MT** | **Sign.** | **pre-MTP** | **post-MTP** | **Sign.** |
| **LOS (days) survivors at MBTK per discharge destination** | | | | | | |
| *Subgroup N=217* | *N=55* | *N=162* |  | *N=58* | *N=159* | *P value* |
|  |  |  |  |  |  |  |
| *home/duty/int.* | 19 ± 14 (14) | 11 ± 10 (7) | <0.05 (*) | 9 ± 9 (5) | 13 ± 12 (9) | NS (*) |
| *Local/Hero/ANA* | 9 ± 10 (7) | 7 ± 9 (5) | NS (*) | 6 ± 5 (4) | 8 ± 11 (6) | NS (*) |
| *Role 3 MTF* | 3 ± 6 (1) | 4 ± 8 (1) | NS (*) | 5 ± 9 (1) | 3 ± 7 (1) | NS (*) |
|  |  |  |  |  |  |  |
| **Discharge destination survivors after treatment** | | | | | | |
| *Subgroup N=227* | *N=58* | *N=169* |  | *N=60* | *N=167* |  |
|  |  |  |  |  |  |  |
| *Local - Home‎/Family* | 17.2% | 29% | <0.05 (†) | 21.7% | 27.5% | <0.05 (†) |
| *Returned to duty* | 0.0% | 3% |  | 1.7% | 2.4% |  |
| *International- Home/ hosp.* | 6.9% | 5.9% |  | 3.3% | 7.2% |  |
|  |  |  |  |  |  |  |
| *Local Hospital – TK* | 24.1% | 36.1% |  | 51.7% | 26.3% |  |
| *Local Hospital – Other* | 1.7% | 1.2% |  | 1.7% | 1.2% |  |
| *Camp Hero‎/ANA Hospital* | 10.3% | 5.3% |  | 3.3% | 7.8% |  |
|  |  |  |  |  |  |  |
| *Role 3 MTF – KAF* | 29.3% | 13.6% |  | 8.3% | 21.0% |  |
| *Role 3 MTF – Bastion* | 5.2% | 1.8% |  | 5.0% | 1.8% |  |
| *Other Role 3 MTF* | 5.2% | 4.1% |  | 3.3% | 4.8% |  |
